# Special Hospitals: V. Ophthalmic and Miscellaneous—Cost of Management

**Published:** 1893-09-30

**Authors:** 


### THE INSTITUTIONAL WORKSHOP.

### HOSPITAL ADMINISTRATION.

### V.—SPECIAL HOSPITALS: OPHTHALMIC AND MISCELLANEOUS. — COST OF MANAGEMENT.*

OPHTHALMIC HOSPITALS.

Many have contended that at the present day, when so much attention is devoted to the diseases of the eye in general hospitals, most of which have special ophthalmic departments with a considerable number of beds, it would be well to discontinue this group of special hospitals altogether. Mr. Brudenell Carter, the eminent ophthalmic surgeon, who is a member of the staff of St. George's Hospital, and who was formerly surgeon at the Royal South London Ophthalmic Hospital, holds this opinion very strongly. From inquiries we have made we are inclined to think that the number of ophthalmic beds provided in several general hospitals is far too few for the requirements of the population, and, when all the circumstances are fairly considered, that this class of institution fulfils a useful purpose, and its continuance is desirable in the public interest. Patients suffering from eye diseases very frequently enjoy good bodily health, and in consequence they are able to be up and about during the greater part of each day. The maintenance of their bodily health necessitates a large amount of day-room space, which it is very difficult, if not impossible, to provide at a general hospital. Hence the maintenance of ophthalmic hospitals is desirable, at any rate for the present, and far more accommodation must be provided at general hospitals before the well-being of the public will warrant their abolition.

There are five metropolitan ophthalmic hospitals, all of which are named in the above table.

As in the case of lying-in hospitals, we are of opinion that 15 per cent. is the maximum sum which may properly be expended upon management at this class of institution. If ophthalmic hospitals are to continue to prosper, the managers must take steps to reduce the cost of administration in very many cases, as the figures in the above table conclusively prove. Thus, with one exception, the average cost of management at a metropolitan hospital exceeds the proper maximum outlay under this head by nearly 10 per cent. This is a very grave state of affairs, which we commend to the immediate and earnest attention of the authorities concerned.

The Royal London at Moorfields is able to maintain 100 beds, of which 83 are constantly occupied, and to treat upwards of 20,000 out-patients without expending more than 12 per cent. on administration compared with maintenance. Further, the percentage of the cost of administration to the total ordinary expenditure is only a little over 10½ per cent. Here we have clear evidence of the justice of maintaining that 15 per cent. is the maximum sum which any ophthalmic hospital ought to expend upon management.

We are glad to see that the Royal South London has reduced its percentage of administration from 25·12 to 24·546 per cent., and the Western Ophthalmic has reduced the percentage of administration from 27·380 to 25·592 per cent., a fact which we cordially recognise as an evidence that the authorities are steadily endeavouring to reduce the expenditure. It is further satisfactory that the Central London should be enabled to reduce the cost of administration by 1 per cent. during 1892, as compared with 1891. On the other hand, the Royal Westminster has increased its percentage of cost of administration to that of maintenance from 25·261 to 26·190 per cent. These latter figures need explanation, which we hope will be forthcoming. It is fair to add, in the case of the Royal Eye, that the in-patient department was closed during the greater part of 1892, during the erection of the new hospital buildings.

METROPOLITAN OPHTHALMIC HOSPITALS.

| Name of Hospital. | Beds for Occupation. | Average Number Occupied. | Total In-patients Admitted. | Total Out-patients Admitted. | Percentage of cost of Administration to that of Maintenance. | Percentage of Administration to Total Expenditure. |
|---|---|---|---|---|---|---|
| Royal London, Moorfields ... ... ... | 100 | 83 | 1,967 | 26,495 | 11·924 | 10·667 |
| Royal (South London) Eye ... ... ... | | rebuilding | | 9,076 | 24·546 | 19·708 |
| Royal Westminster ... ... ... ... | 30 | 26 | 388 | 8,591 | 26·190 | 20·754 |
| Western Ophthalmic ... ... ... ... | 10 | 2 | 54 | 2,478 | 25·892 | 20·570 |
| Central London ... ... ... ... | 13 | 6 | 190 | 8,668 | 20·136 | 16·761 |

* Previous articles of this series have appeared in THE HOSPITAL, page 333 for August 19th; page 349 for August 26th; page 381 for September 9th; and page 413 for September 23rd.

